# *TERT* promoter mutations are highly recurrent in SHH subgroup medulloblastoma

**DOI:** 10.1007/s00401-013-1198-2

**Published:** 2013-10-31

**Authors:** Marc Remke, Vijay Ramaswamy, John Peacock, David J. H. Shih, Christian Koelsche, Paul A. Northcott, Nadia Hill, Florence M. G. Cavalli, Marcel Kool, Xin Wang, Stephen C. Mack, Mark Barszczyk, A. Sorana Morrissy, Xiaochong Wu, Sameer Agnihotri, Betty Luu, David T. W. Jones, Livia Garzia, Adrian M. Dubuc, Nataliya Zhukova, Robert Vanner, Johan M. Kros, Pim J. French, Erwin G. Van Meir, Rajeev Vibhakar, Karel Zitterbart, Jennifer A. Chan, László Bognár, Almos Klekner, Boleslaw Lach, Shin Jung, Ali G. Saad, Linda M. Liau, Steffen Albrecht, Massimo Zollo, Michael K. Cooper, Reid C. Thompson, Oliver O. Delattre, Franck Bourdeaut, François F. Doz, Miklós Garami, Peter Hauser, Carlos G. Carlotti, Timothy E. Van Meter, Luca Massimi, Daniel Fults, Scott L. Pomeroy, Toshiro Kumabe, Young Shin Ra, Jeffrey R. Leonard, Samer K. Elbabaa, Jaume Mora, Joshua B. Rubin, Yoon-Jae Cho, Roger E. McLendon, Darell D. Bigner, Charles G. Eberhart, Maryam Fouladi, Robert J. Wechsler-Reya, Claudia C. Faria, Sidney E. Croul, Annie Huang, Eric Bouffet, Cynthia E. Hawkins, Peter B. Dirks, William A. Weiss, Ulrich Schüller, Ian F. Pollack, Stefan Rutkowski, David Meyronet, Anne Jouvet, Michelle Fèvre-Montange, Nada Jabado, Marta Perek-Polnik, Wieslawa A. Grajkowska, Seung-Ki Kim, James T. Rutka, David Malkin, Uri Tabori, Stefan M. Pfister, Andrey Korshunov, Andreas von Deimling, Michael D. Taylor

**Affiliations:** 1 The Arthur and Sonia Labatt Brain Tumour Research Centre, The Hospital for Sick Children, Toronto, ON Canada; 2 Developmental and Stem Cell Biology Program, The Hospital for Sick Children, Toronto, ON Canada; 3 Department of Laboratory Medicine and Pathobiology, University of Toronto, Toronto, ON Canada; 4 Department of Neuropathology, University Hospital Heidelberg, Heidelberg, Germany; 5 Clinical Cooperation Unit Neuropathology, German Cancer Research Center (DKFZ), Heidelberg, Germany; 6 Division of Pediatric Neurooncology, German Cancer Research Center (DKFZ), Heidelberg, Germany; 7 Department of Pathology, Erasmus Medical Center, Rotterdam, The Netherlands; 8 Department of Neurology, Erasmus Medical Center, Rotterdam, The Netherlands; 9 Departments of Neurosurgery and Hematology and Medical Oncology, School of Medicine and Winship Cancer Institute, Emory University, Atlanta, GA USA; 10 Department of Pediatrics, University of Colorado Denver, Aurora, CO USA; 11 Department of Pediatric Oncology, School of Medicine, Masaryk University and University Hospital Brno, Brno, Czech Republic; 12 Department of Pathology and Laboratory Medicine, University of Calgary, Calgary, AB Canada; 13 Department of Neurosurgery, Medical and Health Science Centre, University of Debrecen, Debrecen, Hungary; 14 Division of Anatomical Pathology, Department of Pathology and Molecular Medicine, McMaster University, Hamilton, ON Canada; 15 Department of Neurosurgery, Chonnam National University Research Institute of Medical Sciences, Chonnam National University Hwasun Hospital and Medical School, Chonnam, South Korea; 16 Department of Pathology, University of Arkansas for Medical Sciences, Little Rock, AR USA; 17 Department of Neurosurgery, David Geffen School of Medicine at UCLA, Los Angeles, CA USA; 18 Department of Pathology, McGill University, Montreal, QC Canada; 19 Dipartimento di Medicina Molecolare e Biotecnologie Mediche, University of Naples, Naples, Italy; 20 CEINGE Biotecnologie Avanzate, Naples, Italy; 21 Department of Neurology, Vanderbilt Medical Center, Nashville, TN USA; 22 Department of Neurological Surgery, Vanderbilt Medical Center, Nashville, TN USA; 23 Laboratoire de Génétique et Biologie des Cancers, Institut Curie, Paris, France; 24 Department of Pediatric Oncology, Institut Curie and University Paris Descartes, Sorbonne Paris Cité, Paris, France; 25 2nd Department of Pediatrics, Semmelweis University, Budapest, Hungary; 26 Department of Surgery and Anatomy, Faculty of Medicine of Ribeirão Preto, Universidade de São Paulo, São Paulo, Brazil; 27 Pediatric Hematology-Oncology, School of Medicine, Virginia Commonwealth University, Richmond, VA USA; 28 Pediatric Neurosurgery, Catholic University Medical School, Rome, Italy; 29 Department of Neurosurgery, Clinical Neurosciences Center, University of Utah, Salt Lake City, UT USA; 30 Department of Neurology, Harvard Medical School, Children’s Hospital Boston, Boston, ME USA; 31 Department of Neurosurgery, Tohoku University Graduate School of Medicine, Sendai, Japan; 32 Department of Neurosurgery, Asan Medical Center, University of Ulsan, Seoul, South Korea; 33 Division of Pediatric Neurosurgery, Department of Neurosurgery, Washington University School of Medicine, St. Louis Children’s Hospital, St. Louis, MO USA; 34 Division of Pediatric Neurosurgery, Department of Neurological Surgery, Saint Louis University School of Medicine, Saint Louis, MO USA; 35 Developmental Tumor Biology Laboratory, Hospital Sant Joan de Déu, Barcelona, Spain; 36 Departments of Pediatrics, Anatomy and Neurobiology, Washington University School of Medicine, St. Louis Children’s Hospital, St. Louis, MO USA; 37 Department of Neurology, University of California, San Francisco, San Francisco, CA USA; 38 Department of Pathology, Duke University, Durham, NC USA; 39 Departments of Pathology, Ophthalmology and Oncology, John Hopkins University School of Medicine, Baltimore, MD USA; 40 Division of Oncology, Cincinnati Children’s Hospital Medical Center, University of Cincinnati, Cincinnati, OH USA; 41 Sanford-Burnham Medical Research Institute, La Jolla, CA USA; 42 Division of Neurosurgery, Department of Surgery, The Hospital for Sick Children and The Arthur and Sonia Labatt Brain Tumour Research Centre, Toronto, ON Canada; 43 Division of Neurosurgery, Hospital de Santa Maria, Centro Hospitalar Lisboa Norte EPE, Lisbon, Portugal; 44 Division of Haematology and Oncology, Department of Pediatrics, The Hospital for Sick Children, University of Toronto, Toronto, ON Canada; 45 Department of Pathology, The Hospital for Sick Children, Toronto, ON Canada; 46 Center for Neuropathology and Prion Research, University of Munich, Munich, Germany; 47 Department of Neurological Surgery, School of Medicine, University of Pittsburgh, Pittsburgh, PA USA; 48 Department of Pediatric Hematology and Oncology, University Medical Center Hamburg-Eppendorf, Hamburg, Germany; 49 Neuro-oncology and Neuro-inflammation Team, Inserm U1028, CNRS UMR 5292, Neuroscience Center, University Lyon 1, 69000 Lyon, France; 50 Centre de Recherche en Neurosciences, INSERM U1028, CNRS UMR5292, Université de Lyon, Lyon, France; 51 Division of Experimental Medicine, McGill University, Montreal, QC Canada; 52 Department of Oncology, The Children’s Memorial Health Institute, Warsaw, Poland; 53 Department of Pathology, The Children’s Memorial Health Institute, Warsaw, Poland; 54 Division of Pediatric Neurosurgery, Department of Neurosurgery, Seoul National University Children’s Hospital, Seoul, Korea; 55 Department of Pediatric Oncology, Hematology, Immunology and Pulmonology, University Hospital Heidelberg, Heidelberg, Germany; 56 Hospices Civils de Lyon, Centre de Pathologie et de Neuropathologie Est, Lyon, 69003 France; 57 Department of Neurology and Neurological Sciences, Stanford University School of Medicine, Stanford, CA USA

**Keywords:** TERT promoter mutations, SHH pathway, Adult, Medulloblastoma

## Abstract

*Telomerase reverse transcriptase* (*TERT*) promoter mutations were recently shown to drive telomerase activity in various cancer types, including medulloblastoma. However, the clinical and biological implications of *TERT* mutations in medulloblastoma have not been described. Hence, we sought to describe these mutations and their impact in a subgroup-specific manner. We analyzed the *TERT* promoter by direct sequencing and genotyping in 466 medulloblastomas. The mutational distributions were determined according to subgroup affiliation, demographics, and clinical, prognostic, and molecular features. Integrated genomics approaches were used to identify specific somatic copy number alterations in *TERT* promoter-mutated and wild-type tumors. Overall, *TERT* promoter mutations were identified in 21 % of medulloblastomas. Strikingly, the highest frequencies of *TERT* mutations were observed in SHH (83 %; 55/66) and WNT (31 %; 4/13) medulloblastomas derived from adult patients. Group 3 and Group 4 harbored this alteration in <5 % of cases and showed no association with increased patient age. The prognostic implications of these mutations were highly subgroup-specific. *TERT* mutations identified a subset with good and poor prognosis in SHH and Group 4 tumors, respectively. Monosomy 6 was mostly restricted to WNT tumors without *TERT* mutations. Hallmark SHH focal copy number aberrations and chromosome 10q deletion were mutually exclusive with *TERT* mutations within SHH tumors. *TERT* promoter mutations are the most common recurrent somatic point mutation in medulloblastoma, and are very highly enriched in adult SHH and WNT tumors. *TERT* mutations define a subset of SHH medulloblastoma with distinct demographics, cytogenetics, and outcomes.

**Electronic supplementary material:**

The online version of this article (doi:10.1007/s00401-013-1198-2) contains supplementary material, which is available to authorized users.

## Introduction

Medulloblastoma is a highly malignant embryonal brain tumor located in the posterior fossa [6, 29, 33, 35]. While this tumor comprises the most common malignant brain tumor in children, it only accounts for approximately 1 % of primary CNS tumors in adults [18, 20]. The current consensus recognizes four core molecular subgroups (WNT, SHH, Group 3, and Group 4) with distinct molecular, demographic, clinicopathological, and prognostic characteristics [5, 15, 16, 26, 27, 37, 38, 41, 42]. The defining features of medulloblastoma subgroups differ dramatically according to age at diagnosis [15, 27, 41]. Specifically, Group 3 tumors are largely confined to non-adults, SHH tumors are most frequent in infants and adults, while WNT and Group 4 medulloblastomas are mostly observed in pediatric cohorts [15, 24, 27, 38, 41]. Particularly within SHH tumors, age-associated heterogeneity was observed regarding the transcriptional characteristics, somatic copy number alterations (SCNA), and the prognostic implications of biomarkers [15, 18, 38, 40]. Delineation of tumorigenic features characteristic for these age-related differences, particularly within SHH tumors, are highly desirable to understand these clear biological and prognostic discrepancies.

Telomere maintenance is fundamentally important to normal self-renewing stem cells and cancer cells [3, 7, 9, 14, 22]. It has been suggested that tumors derived from cell populations with low self-renewal capacity generally rely on alterations that restore telomerase activity, while epigenetic mechanisms maintain telomerase activity in tumor types derived from self-renewing stem cells [13]. The identification of recurrent *telomerase reverse transcriptase* (*TERT*) promoter mutations in 21 % of 91 medulloblastomas [13] is intriguing, since other mechanisms converging on increased telomerase activity including alternative lengthening of telomeres (ALT) [8] or mutations affecting the *ATRX/DAXX* complex are excessively uncommon in medulloblastoma [12, 25, 32, 34, 39]. Although *TERT* mutations have been reported in several cancers [2, 10, 11, 13, 19, 43], their putative association with distinct biological behavior and clinical or even prognostic characteristics has not been comprehensively studied. The initial analyses of *TERT* mutations in medulloblastoma [12] mainly catalogued the mutational frequency rather than correlating the molecular and clinical features of these mutations in a subgroup-specific manner.

In this study, we analyzed a representative set of 466 medulloblastomas for *TERT* promoter mutations. Subsequently, we correlated the mutational distribution with clinicopathological features, outcome, and molecular characteristics in a subgroup-specific manner. We demonstrate that *TERT* promoter mutations comprise the most recurrent mutation in adult SHH tumors identified to date and potentially define distinct prognostic subgroups in SHH and Group 4 medulloblastoma patients.

## Materials and methods

### Tumor material and patient characteristics

All tissues and clinicopathological information were serially collected in accordance with institutional review boards from various contributing centers to this study. Nucleic acid extractions were carried out as previously described [28]. The clinicopathological characteristics of the investigated patient cohort are outlined in Table 1. The median follow-up was 44.06 months (range 0.7–301.5 months).

**Table 1:** Clinicopathological and molecular characteristics according to *TERT* mutational status

Characteristic	*TERT* MUT	*TERT* WT	*p* value
Age (years)			
Median	22.00	7.08	**<0.0001** ^#^
Range	0.66–49.00	0.24–56.32	
NA	1	0	
Gender			
Male	56	236	0.47^Φ^
Female	37	129	
NA	3	5	
Histology			
MBEN	3	8	0.59^χ^
Desmoplastic	10	59	
Classic	46	217	
LC/A	11	38	
NA	26	48	
M-stage			
M0	58	240	**0.03** ^Φ^
M1-3	12	103	
NA	26	27	
*TP53* status			
MUT	4	12	0.78^Φ^
WT	42	97	
NA	50	261	
Subgroup			
WNT	6	47	**<0.0001** ^χ^
SHH	80	133	
Group 3	2	48	
Group 4	8	142	

*F* female, *LC/A* large cell/anaplastic, *M* male, *MB* medulloblastomal, *MBEN* medulloblastoma with extensive nodularity, *NA* not available (data were excluded from statistical comparison)

Bold values indicate *p* < 0.05

^#^Mann–Whitney *U* test

^Φ^Fisher’s exact test

^χ^Chi-square test

### Gene expression and copy number analysis

Subgroup affiliation was determined using nanoString limited gene expression profiling as previously described [31]. Somatic copy number alterations were assessed on the Affymetrix Single Nucleotide Polymorphism (SNP) 6.0 array platform in 418 of 466 cases to identify SCNAs specific for *TERT* mutant and wild-type tumors. Raw copy number estimates were obtained in dChip, followed by CBS segmentation in R as previously described [30]. Somatic copy number alterations were identified using GISTIC2 [21]. *TERT* expression levels were compared using R2 (www.r2.amc.nl). Differences in expression were tested using one-way ANOVA.

### Sanger sequencing

Isolated DNA (25 ng) from all 466 tumors and 7 matched germline samples (25 ng) was amplified by PCR. PCRs contained 1 μl DNA template, 10 μM forward (5′-CAG GGC ACG CAC ACC AG-3′) and reverse (5′-GTC CTG CCC CTT CAC CTT C-3′) TERT-specific primers, and 12.5 μl HotStar Taq Plus Master Mix (Qiagen, Gaithersburg, Maryland, USA) in a 25 μl total reaction volume. Cycle parameters comprised 95 °C × 15 min; 28 cycles of 98 °C × 40 s, 65 °C × 30 s, 72 °C × 1 min; 72 °C × 10 min. PCRs were carried out using the C1000 Thermal Cycler (BioRad, Hercules, CA, USA). PCR products were purified with the PureLink PCR Micro kit (Life Technologies, Burlington, ON, Canada). In all experiments, controls were included in the absence of DNA to rule out contamination by PCR products. Templates for Sanger sequencing were analyzed with forward (5′-CAG CGC TGC CTG AAA CTC-3′) and reverse (5′-GTC CTG CCC CTT CAC CTT C-3′) sequencing primers using dGTP BigDye Terminator v3.0 Cycle Sequencing Ready Reaction Kit (Life Technologies), and 5 % DMSO on the ABI3730XL capillary genetic analyzer (Life Technologies).

### Genotyping assay

Two primers (forward primer, 5′-CAG CGC TGC CTG AAA CTC-3′; reverse primer, 5′-GTC CTG CCC CTT CAC CTT C-3′) were designed to amplify a 163-bp product encompassing C228T and C250T hotspot mutations in the *TERT* promoter—corresponding to the positions 124 and 146 bp, respectively, upstream of the ATG start site. Two fluorogenic LNA probes were designed with different fluorescent dyes to allow single-tube genotyping. One probe was targeted to the WT sequence (*TERT* WT, 5′-5HEX-CCC CTC CCG G-3IABkFQ-3′), and one was targeted to either of the two mutations (*TERT* mut, 5′-56FAM-CCC CTT CCG G-3IABkFQ). Primer and probes were custom designed by Integrated DNA Technologies (Coralville, Iowa, USA) using internal SNP design software, and sequence homogeneity was confirmed by comparison to all available sequences on the GenBank database using BLAST (http://www.ncbi.nlm.nih.gov/BLAST/). Primers were optimized to avoid for hairpins and homo- and heterodimers. Primers and probes were obtained from Integrated DNA Technologies.

Real-time PCR was performed in 25 μl reaction mixtures containing 12.5 μl of TaqMan Universal Master Mix II with UNG (Applied Biosystems), 900 nM concentrations of each primer, 250 nM *TERT* WT probe, 250 nM *TERT* MUT probe, and 1 μl (25 ng) of sample DNA. Thermocycling was performed on the StepOnePlus (Applied Biosystems) and consisted of 2 min at 50 °C, 10 min at 95 °C, and 40 cycles of 95 °C for 15 s and 60 °C for 1 min.

Analysis was performed using StepOne Software, version 2.1. Samples were considered mutant if they had CT values of ≤39 cycles. Each sample was verified visually by examining the PCR curves generated to eliminate false positives due to aberrant light emission. End-point allelic discrimination genotyping was performed by visually inspecting a plot of the fluorescence from the WT probe versus the MUT probe generated from the post-PCR fluorescence read.

### Statistical analysis

Survival time according to *TERT* mutational status was assessed using the Kaplan–Meier estimate and a log-rank test. Comparisons of binary and categorical patient characteristics between subgroups and cohorts were performed using the two-sided Fisher’s exact test or Chi-squared test. Continuous variables were analyzed using the Mann–Whitney *U* test. *p* values <0.05 were considered statistically significant. Multivariate Cox proportional hazards regression was used to adjust for additional covariates using the survival R package (v.2.36). All other statistical analyses were performed using StataSE 12 (Stata Corp. College Station, TX, USA) and Graphpad Prism 5 (La Jolla, CA, USA).

## Results

### Characteristics of *TERT*-mutated medulloblastomas

**Fig. 1:**
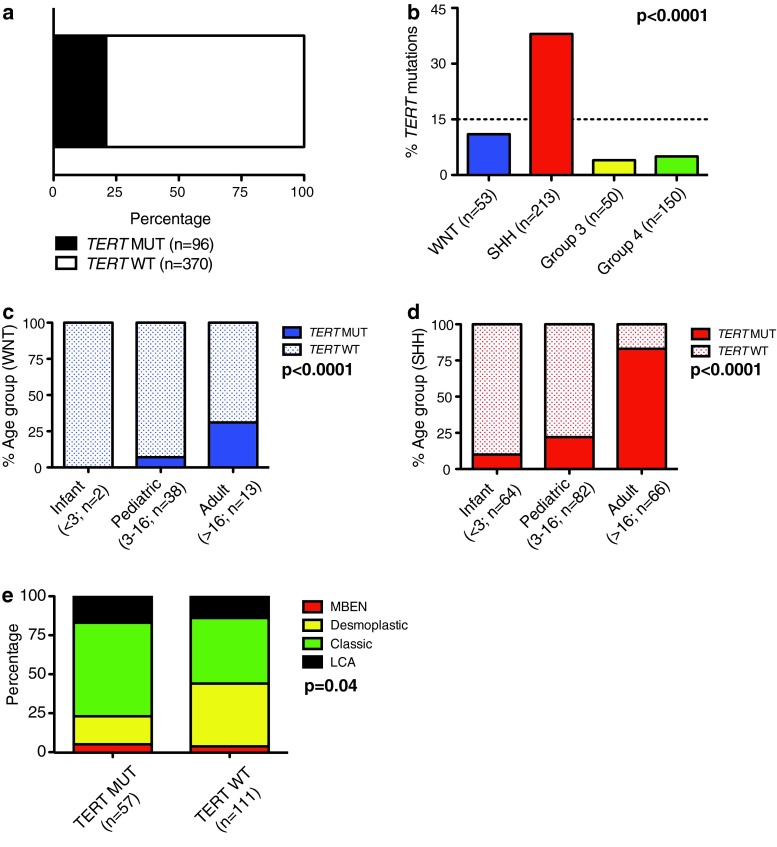
*TERT* promoter-mutated medulloblastomas display distinct demographics, histology, and subgroup affiliation. **a** *Bar graph* indicating the frequency of *TERT* mutations in 466 primary medulloblastomas. **b** Prevalence of *TERT* mutations according to medulloblastoma subgroups, and within, **c** WNT and **d** SHH subgroups according to age groups. Distribution of histological variants within SHH tumors according to *TERT* mutational status (**e**). *MUT* mutation, *OS* overall survival, *WT* wild-type

We performed Sanger sequencing on a clinically well-annotated medulloblastoma cohort (*n* = 466), reflecting the spectrum of demographics and histological subtypes of the disease (Table 1; Supplementary Figure 1A). Our results were verified using a Taqman-based genotyping assay that detects both of the most highly recurrent *TERT* promoter mutations (C228T and C250T). Since both mutational hotspots are located in highly homologous sequences, C228T and C250T mutations result in an identical binding sequence for the mutation-specific probe (CCCGG**A**AGGGG; Supplementary Figure 1B). A total of 21 % of medulloblastomas harbored *TERT* mutations (Fig. 1a). In line with a previous report, these mutations were enriched in older patients (Table 1; *p* < 0.0001), all mutations were heterozygous, and none of the available matched germline controls displayed this mutation [13]. Interestingly, we found that *TERT*-mutated medulloblastomas present less frequently with metastatic dissemination at diagnosis compared to *TERT* wild-type tumors (*p* = 0.03).

### *TERT* mutations are specifically enriched in SHH medulloblastomas

In a subgroup-specific analysis, we revealed that *TERT* mutations were significantly enriched in SHH tumors (80/213; 38 %; *p* < 0.0001) compared to WNT (6/53; 11 %) and Group 3 (2/50; 4 %) or Group 4 tumors (8/150; 5 %). *TERT* mutations in both WNT and SHH medulloblastomas were positively correlated with age. *TERT* mutations were significantly enriched in adult patients (Fig. 1c, d, both *p* < 0.0001). Increasing age was not associated with increased mutational frequency in either Group 3 or Group 4 tumors (n.s.). While histopathological features were similar between *TERT*-mutated and wild-type tumors across subgroups, we observed that classic histology was more commonly observed in *TERT* mutant SHH tumors, and desmoplastic histology in wild-type SHH tumors (Fig. 1e; Table 2; *p* = 0.04), respectively.

**Table 2:** Clinicopathological and molecular characteristics of SHH medulloblastoma according to *TERT* mutational status

Characteristic	*TERT* MUT	*TERT* WT	*p* value
Age (years)			
Median	25.00	3.00	**<0.0001** ^#^
Range	0.66–49.00	0.24–52.00	
NA	1	0	
Gender			
Male	46	80	0.77^Φ^
Female	31	48	
NA	3	5	
Histology			
MBEN	3	4	**0.04** ^χ^
Desmoplastic	10	44	
Classic	34	47	
LC/A	10	16	
NA	23	22	
M-stage			
M0	46	87	0.84^Φ^
M1-3	10	22	
NA	24	24	
*TP53* status			
MUT	4	8	1^Φ^
WT	38	71	
NA	38	54	

*F* female, *LC/A* large cell/anaplastic, *M* male, *MB* medulloblastoma, *MBEN* medulloblastoma with extensive nodularity, *NA* not available (data were excluded from statistical comparison)

Bold values indicate *p* < 0.05

^#^Mann–Whitney *U* test

^Φ^Fisher’s exact test

^χ^Chi-square test

### Prognostic implications of *TERT* mutations

**Fig. 2:**
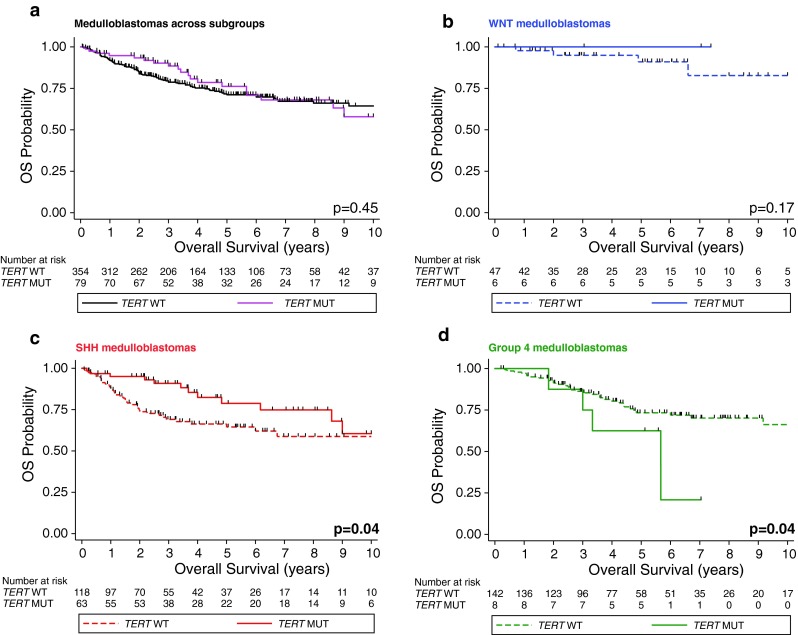
Prognostic impact of *TERT* promoter mutations varies according to medulloblastoma subgroups. Kaplan–Meier estimate displaying overall survival (OS) according to *TERT* mutational status in primary medulloblastomas (**a**), within WNT (**b**), SHH (**c**), and Group 4 (**d**) subgroups. Survival differences were calculated using continuous log-rank tests. *MUT* mutation, *OS* overall survival, *WT* wild-type

When medulloblastoma patients across all subgroups were stratified by *TERT* mutational status, we observed no significant differences in survival (Fig. 2a; *p* = 0.45). Further after normalizing the subgroup composition to reported subgroup ratios, a statistical difference was still not revealed (data not shown; *p* = 0.36) [1, 15, 26, 41]. However, when *TERT* mutational status is re-analyzed in a subgroup-specific manner, several important survival associations are observed. *TERT* mutations had no prognostic impact within WNT tumors (Fig. 2b; *p* = 0.17). However, a significant association between *TERT* promoter mutations and outcomes was noted in SHH and Group 4 medulloblastomas. Specifically, the 5-year overall survival of SHH tumors with and without *TERT* mutations was 77.6 ± 7 % and 64.1 ± 5.1 %, respectively (Fig. 2c; *p* = 0.04). In contrast to the improved prognosis of *TERT* mutant SHH tumors, we observed the inverse pattern in Group 4 tumors where the 5-year overall survival for patients without and with *TERT* mutations was 73.3 % ± 4.3 % and 62.5 % ± 17.1 % (Fig. 2d; *p* = 0.04). Similar to the unfavorable prognosis of *TERT* mutations in Group 4 tumors, both of the patients with *TERT*-mutated Group 3 tumors died after 7 and 45 months of follow-up, respectively (Supplementary Table 1). Thus, we conclude that *TERT* mutations define distinct prognostic patient cohorts in a subgroup-specific fashion with good prognosis in SHH and poor prognosis in Group 4 medulloblastomas.

### Survival analysis restricted to specific age groups

**Fig. 3:**
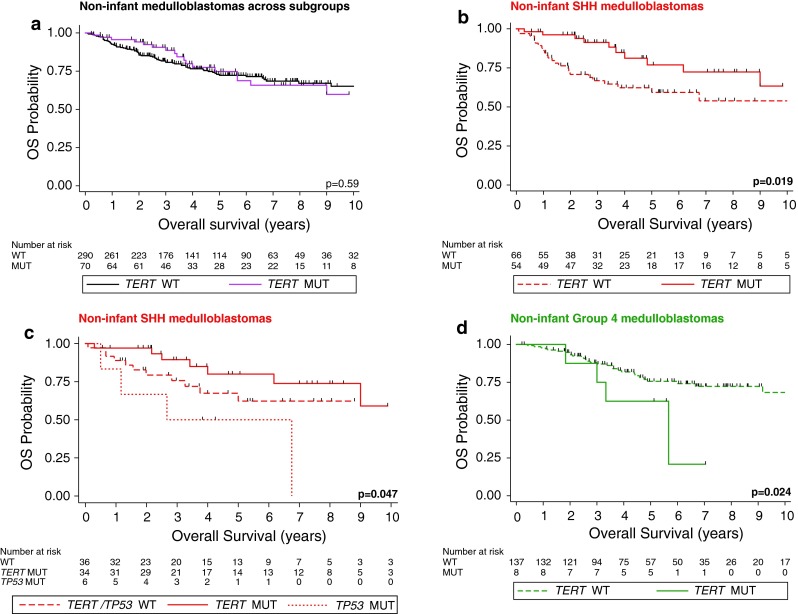
*TERT* promoter mutations delineate prognostic subsets within non-infant SHH and Group 4 medulloblastomas. Kaplan–Meier estimate displaying overall survival (OS) in non-infant medulloblastomas (>3 years of age at diagnosis) according to *TERT* mutational status across subgroups (**a**), in SHH tumors (**b**), in SHH tumors (*TP53* mutated/wild-type) (**c**), and Group 4 (**d**). Survival differences were calculated using continuous log-rank tests

As *TERT* mutations are predominantly observed in non-infant medulloblastomas, we evaluated the prognostic implications of these promoter mutations across all four medulloblastoma subgroups in an age-dependent manner. *TERT* mutational status across subgroups had no prognostic impact among patients older than 3 years of age at diagnosis (Fig. 3a; *p* = 0.59). Interestingly, the prognostic impact of *TERT* mutation was more pronounced in the non-infant SHH population with a 5-year overall survival of 76.9 % ± 7.6 % and 59.3 % ± 6.9 % of non-infants with and without *TERT* promoter mutations, respectively (Fig. 3b; *p* = 0.019). These prognostic implications were similar in adult medulloblastoma patients and in the adult SHH subgroup (Supplementary Figure 2). In a subset of 76 SHH cases with known *TP53* mutational status [44], we revealed that *TP53* mutations identify non-infant SHH tumors with a particularly poor prognosis, while in contrast *TERT* mutations identify a subsets with good prognosis (Fig. 3c; *p* = 0.047). Mutations of both *TERT* and *TP53* were observed in 4/12 SHH tumors (Supplementary Table 2). Non-infant Group 4 showed an inverse prognostic association with poor outcome of *TERT*-mutated cases (Fig. 3d; *p* = 0.024). Lastly, we analyzed the overall survival of SHH patients under a multivariate Cox proportional hazards model comprising age at diagnosis, *TERT* mutational status, M-stage, and histology. In addition to the known prognostic significance of M-stage (*p* < 0.001) and histology (*p* = 0.02), we revealed that *TERT* status continued to be associated with good prognosis (HR 0.17, CI 0.04–0.69, *p* = 0.01), independent of other prognostic factors including age at diagnosis (*p* = 0.35).

### Distinct somatic copy number alterations of *TERT*-mutated medulloblastomas

**Fig. 4:**
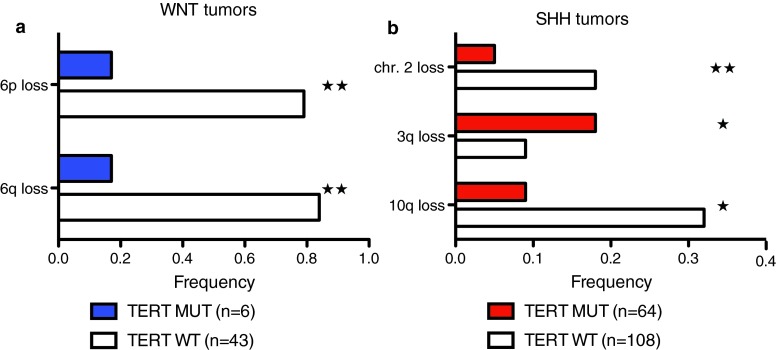
WNT and SHH medulloblastoma harbor distinct broad genomic imbalances depending on the mutational status of *TERT*. *Bar graphs* indicating the frequency of broad cytogenetic alterations in WNT (**a**), and SHH (**b**) tumors. ★★ *p* < 0.01; ★ *p* < 0.05; *MUT* mutation, *WT* wild-type

**Fig. 5:**
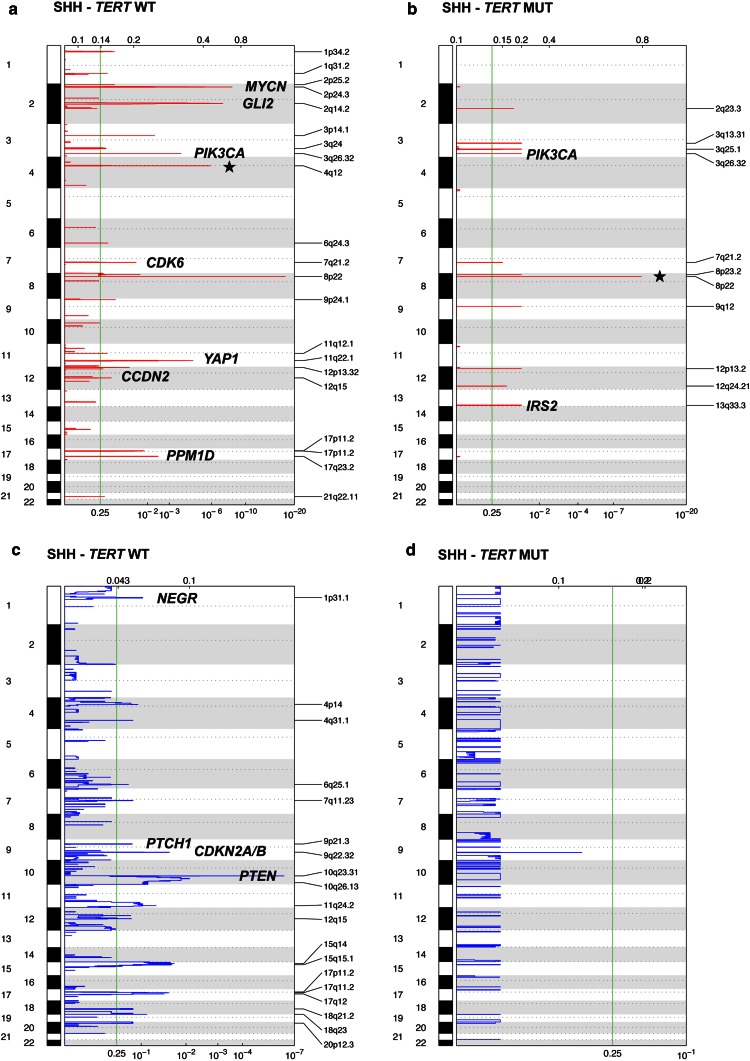
Focal somatic copy number alterations are largely confined to *TERT* wild-type SHH medulloblastomas. GISTIC2 analysis indicating focal amplifications/deletions in 108 wild-type (**a**, **c**) and 64 mutant (**b**, **d**) SHH tumors, respectively. *Star* regions enriched for reported DNA copy number variations

To identify additional genetic features associated with these distinct demographic and clinical differences, we evaluated broad and focal copy number alterations according to subgroup affiliation and *TERT* promoter mutations. Notably, only 1/6 (17 %) of *TERT*-mutated WNT tumors harbored monosomy 6, while this alteration is observed in approximately 80 % of *TERT* wild-type medulloblastomas of the WNT subgroup (Fig. 4a; *p* = 0.005). Loss of chromosome 2 and 10q loss were significantly enriched in *TERT* wild-type SHH tumors, while 3q loss was more frequently observed in their *TERT* mutant counterparts (Fig. 4b). Previously described focal alterations characteristic for SHH tumors including amplification of *MYCN/GLI2/CDK6/YAP1/PPM1D*, and deletions targeting *PTCH1/CDKN2A/CDKN2B/PTEN* were largely confined to *TERT* wild-type SHH medulloblastomas, while *TERT* mutant SHH (Fig. 5) and Group 4 (Supplementary Figure 3) showed very few focal SCNAs. Consistent with the higher frequency of *TERT* mutations in SHH tumors, we observed increased *TERT* expression in the SHH subgroup compared to Group 4 tumors in two independent gene expression profiling studies (*p* < 0.001; Supplementary Figure 4). Furthermore, we observed *TERT* amplification in two tumors included in the entire cohort of 1,088 previously studied tumors [30]. Both of these cases with *TERT* amplification were SHH-driven medulloblastomas with wild-type *TERT* status, which were derived from pediatric patients who were both alive after 15 and 83 months of follow-up (Supplementary Figure 5). Thus, broad and focal SCNAs underline that *TERT* mutations define a genetically distinct subset within SHH tumors and possibly within the WNT and Group 4 tumors.

## Discussion

The underlying biology of adult medulloblastomas remains poorly understood. Next-generation sequencing studies have revealed a broad spectrum of novel, potentially tumorigenic mutations in the recent past, but none of these studies focused on adult medulloblastomas [12, 25, 32, 34, 39]. In addition, the vast majority of these mutations are not recurrent enough to stratify patients into distinct clinical and prognostic subgroups.

In this study, we demonstrate that *TERT* promoter mutations, initially described in melanoma [10, 11], comprise the most recurrent mutation described so far across medulloblastoma subgroups, with a particular enrichment in older patient cohorts. These somatic mutations are especially common in older patients with SHH tumors (83 %) and to a lesser extent in adults with WNT medulloblastomas (11 %). Based on the transcriptional heterogeneity of SHH tumors in infant and adult patients, we suspect that the adult cluster mainly comprised *TERT*-mutated medulloblastomas [24]. According to the initial classification of tumor types with *TERT* mutations at frequencies over 15 % (*TERT*-high) vs. below this threshold (*TERT*-low) [13], our report suggests distinct baseline telomerase activity of the cell of origin in each of the subgroups (Group 3 ≥ Group 4 > WNT >> SHH). Furthermore, the identification of recurrent *TERT* promoter mutations makes a compelling argument that the increasing availability of whole-genome sequencing results may substantially add to a refined understanding of the mutational landscape of different biological and age-driven medulloblastoma subgroups, since earlier next-generation sequencing studies focusing on the protein-coding regions had not encompassed gene-regulatory regions including promoter mutations.

In this study, we demonstrate that the mutational status of the *TERT* promoter can segregate individuals with SHH and Group 4 medulloblastomas with distinct prognostic outcomes, while a prognostic impact of this mutation was not observed in glioblastomas [23]. Molecular mechanisms converging on *TERT* up-regulation were recently reported to be associated with dismal prognosis in pediatric brain cancers [4]. Our findings in Group 4 tumors with *TERT* mutations follow this pattern, while SHH tumors with *TERT* mutations comprise a prognostically favorable subgroup. Notably, survival curves of SHH tumors increasingly approximate with extended follow-up. We hypothesize that this pattern might be due to secondary malignancies and late relapses in older SHH tumors [36–38]. Since virtually all of the *TERT* promoter mutations encompass the mutational hotspots C228T and C250T, patient stratification can be carried out using a single PCR followed up with Sanger sequencing or with a single experiment using our newly designed Taqman-based genotyping assay. The latter assay is particularly suitable for routine clinical applications as it is highly sensitive and specific (5 ng DNA input is sufficient). Furthermore, our Taqman-based genotyping assay can be used on DNA derived from fresh-frozen and formalin-fixed paraffin-embedded tissue, since it only amplifies a short DNA fragment.

Both hotspot mutations C228T and C250T create an E-twenty-six (ETS) binding motif [10, 11] resulting in up-regulation of *TERT* expression at the mRNA level [2], which was not observed at the protein level in glioblastomas [43]. We now demonstrate that SHH tumors with *TERT* mutations are mostly mutually exclusive with those harboring 10q loss (*p* = 0.017) Notably, the relatively favorable prognosis of *TERT*-mutated SHH medulloblastomas may be explained by the relative lack of high-risk biomarkers [17, 18, 24, 44].

In summary, we describe the demographic, clinicopathological, and biological implications of *TERT* promoter mutations in a subgroup-specific fashion. This study underlines the dependence of adult WNT and SHH tumors to reacquire telomerase activity and suggests a potential prognostic utility of *TERT* mutational analysis in an era of individualized therapy.

## Electronic supplementary material

Below is the link to the electronic supplementary material.

Representative electropherograms (a) and genotyping results (b) of the wild-type and mutated *TERT* promoter sequence (JPEG 1454 kb)

Overall survival (OS) in adult medulloblastomas (a), and adult SHH-driven tumors (b) according to *TERT* mutational status. Survival differences were calculated using continuous log-rank tests. Abbreviations: MUT, mutation; WT, wild-type (EPS 650 kb)

Focal somatic copy number alterations are largely confined to *TERT* wild-type Group 4 tumors. GISTIC2 analysis indicating focal amplifications/deletions in 140 wild-type (a/c) and 7 mutant (b/d) Group 4 tumors, respectively. Legend: ★, regions enriched for reported DNA copy number variations (JPEG 1392 kb)

Transcriptional profiling reveals high *TERT* expression in SHH medulloblastoma. Boxplots illustrate significantly higher levels of *TERT* expression in SHH tumors compared to Group 4 medulloblastomas (EPS 869 kb)

Minimally overlapping region including the *TERT* gene on chromosome arm 5p. DNA copy number gains and losses are indicated by red and blue, respectively (EPS 21771 kb)

Patient characteristics of non-SHH *TERT*-mutated medulloblastoma (DOCX 153 kb)

Supplementary material 7 (DOCX 3 kb)
