# Supplementary figures and images for: TERT promoter mutations are highly recurrent in SHH subgroup medulloblastoma
Source: Acta Neuropathol. 2013 Oct 31;126(6):917–29. doi: 10.1007/s00401-013-1198-2 (PMC3830749; doi:10.1007/s00401-013-1198-2)

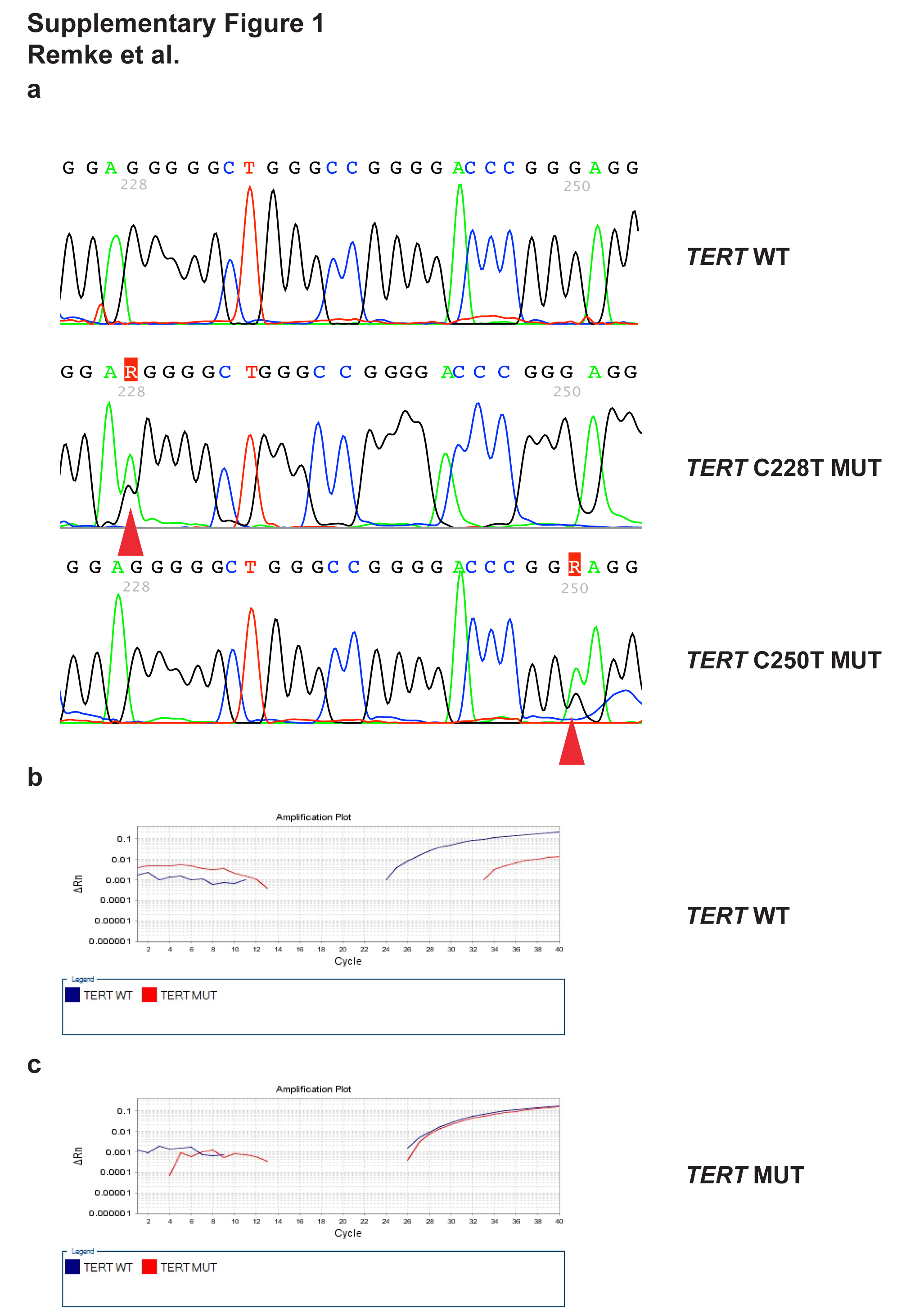

Supplement: Supplementary file 1 — Representative electropherograms (a) and genotyping results (b) of the wild-type and mutated TERT promoter sequence (JPEG 1454 kb) [file 401_2013_1198_MOESM1_ESM.jpg]

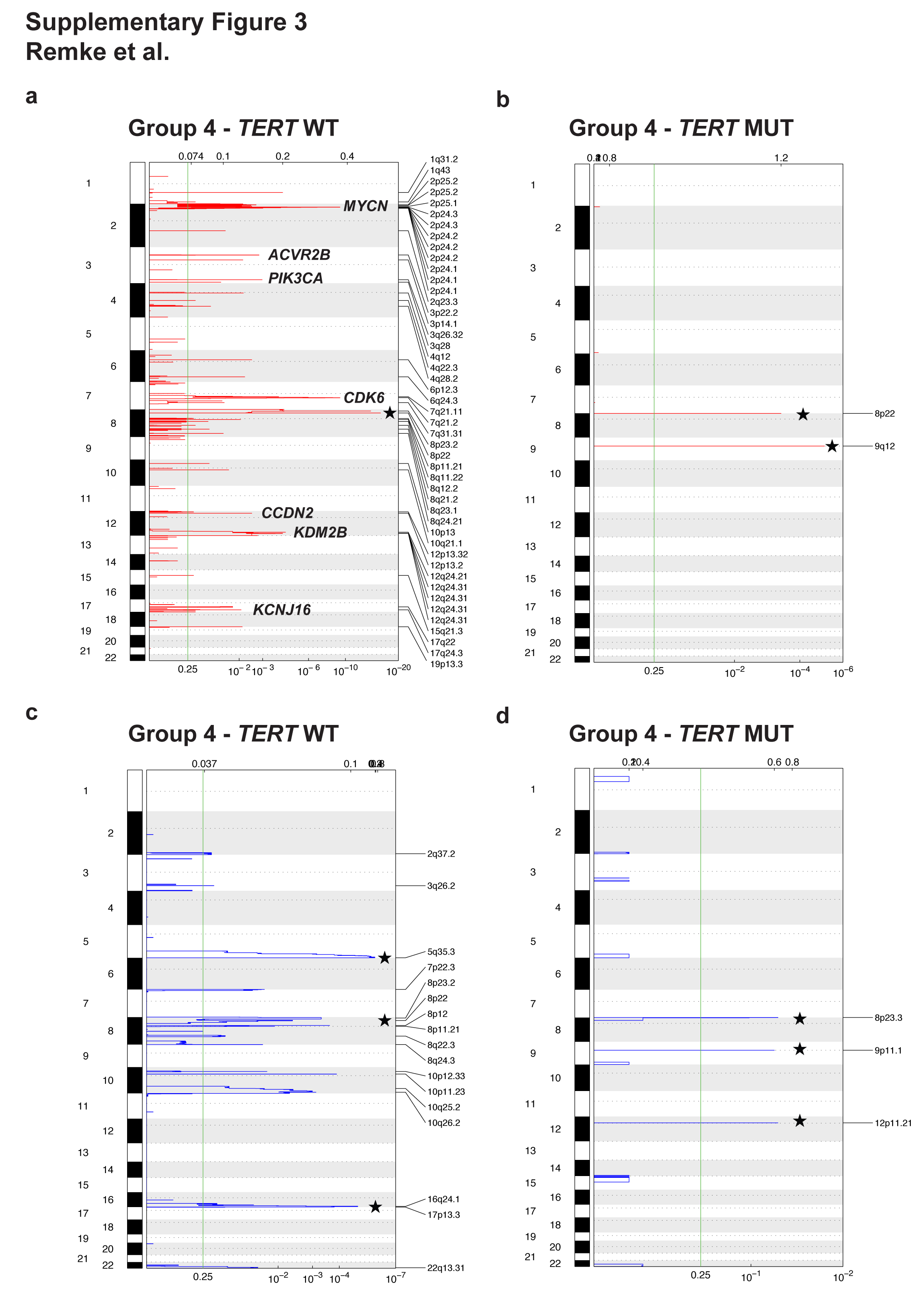

Supplement: Supplementary file 3 — Focal somatic copy number alterations are largely confined to TERT wild-type Group 4 tumors. GISTIC2 analysis indicating focal amplifications/deletions in 140 wild-type (a/c) and 7 mutant (b/d) Group 4 tumors, respectively. Legend: ★, regions enriched for reported DNA copy number variations (JPEG 1392 kb) [file 401_2013_1198_MOESM3_ESM.jpg]
