# Supplementary material for: TERT promoter mutations are highly recurrent in SHH subgroup medulloblastoma
Source: Acta Neuropathol. 2013 Oct 31;126(6):917–29. doi: 10.1007/s00401-013-1198-2 (PMC3830749; doi:10.1007/s00401-013-1198-2)
Supplement: Supplementary file 6 — Patient characteristics of non-SHH TERT-mutated medulloblastoma (DOCX 153 kb) [file 401_2013_1198_MOESM6_ESM.docx]

**Supplementary Table 1. Non-SHH *TERT* mutated medulloblastoma**

| Sample ID | Subgroup | Age (years) | Gender | Histology | OS  (months) |
| --- | --- | --- | --- | --- | --- |
| MDT-MB-31 | WNT | 5 | Female | Classic | Alive (120) |
| MDT-MB-730 | WNT | 24 | Male | Classic | Alive (120) |
| MDT-MB-190 | WNT | 36 | Male | Classic | Alive (120) |
| MDT-MB-1235 | WNT | 20 | Female | Classic | Alive (36) |
| MDT-MB-566 | WNT | 8 | Female | Classic | Alive (88) |
| MDT-MB-416 | WNT | 19 | Female | Classic | Alive (84) |
| MDT-MB-412 | Group3 | 29 | Male | Classic | Dead (45) |
| MDT-MB-511 | Group3 | 5 | Male | MB | Dead (7) |
| MDT-MB-551 | Group4 | 9 | Male | LC/A | Dead (22) |
| MDT-MB-608 | Group4 | 10 | Female | Classic | Dead (40) |
| MDT-MB-210 | Group4 | 12 | Male | Classic | Alive (67) |
| MDT-MB-840 | Group4 | 7 | Male | MB | Dead (68) |
| MDT-MB-379 | Group4 | 6 | Male | Classic | Alive (61) |
| MDT-MB-390 | Group4 | 4 | Male | Classic | Dead (68) |
| MDT-MB-1145 | Group4 | 13 | Male | Classic | Alive (84) |
| MDT-MB-2126 | Group4 | 6 | Female | MB | Dead (36) |

**Abbreviations:** LC/A, large-cell / anaplastic; MB, medulloblastoma.
