# Supplementary material for: TERT promoter mutations are highly recurrent in SHH subgroup medulloblastoma
Source: Acta Neuropathol. 2013 Oct 31;126(6):917–29. doi: 10.1007/s00401-013-1198-2 (PMC3830749; doi:10.1007/s00401-013-1198-2)
Supplement: Supplementary file 7 — Supplementary material 7 (DOCX 3 kb) [file 401_2013_1198_MOESM7_ESM.docx]

**Supplementary Table 2.** SHH medulloblastoma with mutations in *TERT* and *TP53*

| Sample ID | Subgroup | Age (years) | Gender | Histology | OS  (months) |
| --- | --- | --- | --- | --- | --- |
| MDT-AP-0707 | SHH | 33 | NA | LC/A | Alive (27) |
| MDT-AP-1250 | SHH | 26 | Female | Classic |  |
| MDT-AP-0843 | SHH | 6 | Female | MB | Dead (44) |
| MDT-AP-0599 | SHH | 12 | Male | LC/A | Alive (44) |

**Abbreviations:** LC/A, large-cell / anaplastic; MB, medulloblastoma; NA, not available.
